# Determinants of Pro-Poor Growth and Its Impacts on Income Share: Evidence from Ethiopian Time Series Data

**DOI:** 10.1155/2021/6645789

**Published:** 2021-03-02

**Authors:** Gemechu Bekana Fufa

**Affiliations:** Department of Statistics, College of Natural and Computational Sciences, Wollega University, Nekemte, Ethiopia

## Abstract

The growing research interest in the pro-poorness of growth is the main issue today. Reducing economic poverty and inequality through pro-poor growth is the aim of policies in many countries. Pro-poor growth is good for poverty eradication if it can be achieved. Ethiopia is a good example of a country where growth was pro-poor between 1990 and 2018 but the pro-poor growth was reversed in 2016. The paper examined what led to pro-poor growth between 1990 and 2018 and what may have been responsible for the reversal in 2016. Unit root test reveals that all the series are nonstationary at level and stationary at first difference and have one cointegration relation between the variables. The dynamic ordinary least squares method was used to analyze the Ethiopian time series data from World Bank Development Indicators between 1990 and 2018 for the determinant of pro-poor growth. Regression analysis shows that job creation was responsible for the pro-poor growth between 1990 and 2018. The results of the analysis showed that human capital, industrial, and services growth have negative impacts on poorest people, whereas employment and agriculture growth have positive impacts on poorest people. In the richest income group, human capital, and industrial and service growths have positive impacts while agricultural growth and employment have negative impacts.

## 1. Introduction

Growth and poverty reduction have been at the center of the discussion in economic development for a long time now. The authors in [1] asserted that economic growth as well as the pattern of the growth is very important in achieving substantial poverty reduction. In the early discussion on the relationship between growth and poverty in 1950s and 60s, the debate was around trickle down hypothesis because of the belief that as an economy grows, its benefits will pass through the rich down to poor and solve poverty problem [2]. However, experience showed that, in many developing countries, poverty increased instead of reducing with growth, especially in sub-Saharan African countries [3].

Pro-poor growth focuses attention on the extent to which poor women and men are able to participate in, contribute to, and benefit from growth, as measured by changes in the incomes of the households in which they live and the assets they and their children acquire to earn higher incomes in the future. When may growth be termed pro-poor? There are different views on this issue. For some, what matters is whether the incomes of the poor are rising relative to the incomes of the non-poor and hence inequality is falling. The merit of this perspective is that it focuses attention on whether the poor are benefiting more or less proportionately from growth and whether inequality, a key determinant of the extent to which growth reduces poverty, is increasing or falling [4].

The simultaneous rise in growth and poverty in many countries in late 1990s and early 2000 led to a shift in discussions on growth and poverty to pro-poor growth. There are many definitions of pro-poor growth. According to [2], pro-poor growth is the growth which favors the poor more than the non-poor in income redistribution. Similarly, the paper [5] points out that pro-poor growth means the growth in favor of the poor. The authors in [6] defined pro-poor growth as that growth which can increase the share of the poor from growth above the international norm. In the definition given by [7, 8], pro-poor growth in its absolute term is the growth which can reduce poverty, and from the relative term it is the growth which can increase the income of the poor disproportionately such that inequality falls.

Poverty is harmful to growth and development. According to [9], poverty reduction is very essential because its reduction can speed up growth. The paper [10] emphasized that it is good to fight poverty because it is one of the factors which is threatening world growth. De la Fuente maintained that, to increase the speed of world development, effort must be made to improve the current world living standard [10]. The assertion fell in line with the view of the authors in [1] who maintained that poverty impairs growth. Therefore, pursuance of poverty reduction policy will expectedly lead to sustainable economic growth and world development in the twenty-first century. The poor are the people who lack the resources to meet basic needs that improve wellbeing [3]; or, they are the people who have higher risk of disease or people who live in sub-standard conditions and lack basic infrastructure [11]. The authors in [12] defined the poor as the people on the bottom fifth of income distribution.

## 2. Related Works

In the pro-poor growth debate, the paper [7] points out that the poor are mainly in the rural area doing business in the agriculture sector, and to achieve pro-poor growth, government must direct attention to the improvement in agricultural productivity. This argument is supported by [13] that stresses that pro-poor growth strategy should target the agriculture and rural economy where the poor work. The authors in [14, 15] also accept that the promotion of agricultural productivity can enhance pro-poor growth because it is the sector where the majority of the poor carry out their economic activity.

The authors in [2, 16] in different studies discovered that pro-poor growth depends on country because there is no guarantee that every growth will benefit all the poor in a country. Cross-country research in Asia, Europe, Latin America, and sub-Saharan Africa by [17, 18] discovered different effects of growth on the poor. The paper [14] equally discovered that growth affects the poor differently across countries. From a cross-country study in Asia by Pasha and Palanivel, employment opportunity and growth in agriculture significantly lead to pro-poor growth. Other cross-country investigations showed that some other determinants of pro-poor growth are improvement in education and health, control of corruption, financial openness, and financial development [6].

The fall in income share of the bottom 20%, as well as the rise in poverty in Ethiopia in 2016, is a signal that the pro-poor growth experienced between 1990 and 2010 is being reversed [4, 19]. It raises a serious concern because if the trend continues, the gains of economic prosperity the country recorded for two decades will be lost. To see that the trend is not reversed, it is important to investigate the factors that determine pro-poor growth in Ethiopia and advice policy-makers on the measures to take so as to stop the economy from taking more people back to poverty. While the study is being done in Ethiopia, it will serve as a lesson for other emerging economies, especially the sub-Saharan Africans where poverty is endemic [3, 20].

Growth by itself is not necessarily sufficient in Ethiopia. It needs to be sustainable, sustained, and inclusive. Rapid and sustained poverty reduction requires pro-poor growth, i.e., a pace and pattern of growth that enhances the ability of poor women and men to participate in, contribute to, and benefit from growth [9, 21]. Policies therefore need to promote both the pace of economic growth and its pattern, i.e., the extent to which the poor participate in growth as both agents and beneficiaries, as these are interlinked and both are critical for long-term growth and sustained poverty reduction [22]. Pro-poor growth is economic changes which poor women and men are able to participate in, contribute to, and benefit from growth, as measured by changes in the incomes of the households in which they live and the assets they and their children acquire to earn higher incomes in the future. It also focuses on making the poor benefit from growth, increasing the rate of job creation from growth, making growth more effective in reducing poverty, inequality matters, and being inclusive.

The growing research interest in the pro-poorness of growth is the main issue today. Reducing economic poverty and inequality through pro-poor growth is the aim of policies in many countries. However, it is not fully functionated and the patterns of pro-poor growth are not well known. Given its low productivity, increasing the amount of labour and land is the only way to raise production in agriculture. There is a very large gap between poor and non-poor communities in Ethiopia, especially. Hence, identifying the determinants of pro-poor growth and its impacts is sound enough for this study. Thus, this study aims to identify the determinants of pro-poor growth and its impacts on income share in Ethiopia using time series data from 1990 to 2018.

## 3. Materials and Methodology

### 3.1. Data

This study is mainly based on secondary yearly data from Central Statistical Agency (CSA), National Bank of Ethiopia (NBE), Ministry of Finance and Economic Development (MoFED), Ethiopian Revenue and Customs Authority (ERCA), and various publications of International Monetary Fund (IMF) and World Bank (WB) covering the period from 1990 to 2018. This data was used to establish trends in interannual pro-poor growth distribution for the above-mentioned from 1990 to 2018 years period. Since the yearly sample size was 28, not maximum, the author also analyzed the quarterly data for the given sample size as descriptive form.

### 3.2. Methods of Data Analysis

#### 3.2.1. Ordinary Least Squares Methods

Linear regression models find several uses in real-life problems. In econometrics, ordinary least squares (OLS) method is widely used to estimate the parameter of a linear regression model. OLS estimators minimize the sum of the squared errors (a difference between observed values and predicted values). While OLS is computationally feasible and can be easily used while doing any econometrics test, it is important to know the underlying assumptions of OLS regression. This is because a lack of knowledge of OLS assumptions would result in its misuse and give incorrect results for the econometrics test completed. The importance of OLS assumptions cannot be overemphasized. The next section describes the assumptions of OLS regression.

The econometric model identifies functional relationships between income shares and its determinants using ordinary least square model. The OLS approach to multiple linear regressions was introduced by Schultz in 1988 [23]. The OLS technique is the simplest type of estimation procedure used in statistical analyses [24]. OLS is performed in economics (econometrics), political science, and electrical engineering (control theory and signal processing), among many areas of application. The OLS model includes dependent and independent variables. Additionally, each of these variables must be estimated; therefore, the accuracy of the estimation depends on the reality and precision of each data sample. However, to benefit from the refined properties of an OLS estimate, numerous assumptions must be satisfied. In this study, dependent and independent variables were included. The dependent variable is income share, whereas independent variables are human capital development (HC), growth in agriculture (AGR), total employment (EMP), industrial growth (INDG), growth in services (GRS), and effective labour (AL).

The ordinary least squares method of research was adopted in this study because of its simplicity and good properties of best linear unbiased estimates (BLUE). An exogenous growth model shows a long run economic growth which operates within the framework of the neoclassical economists [25]. The Solow–Swan growth model which explains long run economic growth using capital, labour, and technological progress belongs to this class. Accordingly,

(1) $$\begin{array}{c} Y\left(t\right)=K{\left(t\right)}^{\alpha }{\left(A\left(t\right)L\left(t\right)\right)}^{1-\alpha }, \end{array}$$

where *t* is time, 0 < *α* < 1 is elasticity of output accrued to capital, *Y*(*t*) is total output, A is a labour-augmenting technology, and AL is the effective labour [26]. The effective labour (AL) grows at Ŋ + *g* while capital depreciates at *δ*. Hence, the derivative of *K* with respect to time becomes

(2) $$\begin{array}{c} K\prime \left(t\right)=s*Y\left(t\right)-\delta *K\left(t\right). \end{array}$$

The model for this study is income share function using data from Ethiopia. Growth in some sectors of an economy can create jobs and lead to pro-poor growth [22]. The model is designed to capture the impact of such change in some sectors of the economy of Ethiopia on the income share of the lowest 20% group.

(3) $$\begin{array}{c} \text{YS}=f\left(\text{HC,AGR,EMP,INDG,GRS}\right), \end{array}$$

where YS is income share of a group, HC is human capital development, AGR is growth in agriculture, EMP is total employment, INDG is industrial growth, and GRS is growth in services.

Equation (3) captures the behavior of income share by the lowest and highest 20% income group in Ethiopia.

For estimation, equation (4) is transformed; thus,

(4) $$\begin{array}{c} \text{YS}=\alpha +\beta_{1}{\text{HC}}_{t}+\beta_{2}{\text{AGR}}_{t}+\beta_{3}{\text{EMP}}_{t}+\beta_{4}{\text{INDG}}_{t}+\beta_{5}{\text{GRS}}_{t}+e_{t}, \end{array}$$

where *α* is intercept, *β*_1_–*β*_5_ are coefficients, *e* is error, and *t* is time to denote time series.

Equations (4) and (5) can be transformed further as specified below:

(5) $$\begin{array}{c} Y_{it}=\alpha_{i}+\beta_{i}X_{it}+e_{t}, \end{array}$$

where *Y*_*t*_ represents income share of a quintile group in Ethiopia, *X*_*t*_ is made up of all the explanatory variables as described in the model, and *e*_*i*_ is the error term.

### 3.3. Testing Stationarity: Unit Root Test

Before fitting a particular model to time series data, the series must be made stationary. Stationarity in a time series occurs when the mean remains constant and the autocovariances of the series depend on the lags separating the time points. Therefore, the process *Y*_t_ is said to be stationary if

(6) $$\begin{array}{c} E\left(Y_{t}\right)=\mu ,\text{constant for all value of }t\text{ and,} \end{array}$$

(7) $$\begin{array}{c} \mathrm{cov}\left(Y_{t},Y_{t-j}\right)=\gamma_{j},\text{for all }t,j=0,1,2,\ldots ,T. \end{array}$$

Condition (6) means that all *Y*_*t*_ have the same finite mean vector *μ* and (7) requires that the autocovariances of the process do not depend on *t* but just on the period *j* the two vectors *Y*_*t*_ and *Y*_*t-j*_ are apart. Therefore, a process is stationary if its first and second moments are time invariant.

The stationarity of the series is tested by using statistical tests such as Augmented Dickey–Fuller (ADF) test due to [18, 27] and Philips test due to [25].

Consider a simple AR (1) process

(8) $$\begin{array}{c} Y_{t}=\theta Y_{t-1}+X_{t}^{\prime }\delta +\varepsilon_{t}, \end{array}$$

where *X*_*t*_ are optional exogenous regressors which may consist of constant or a constant and trend, *θ* and *δ* are parameters to be estimated, and *ε*_*t*_ is assumed to be white noise. If |*θ*| ≥ 1, *Y*_*t*_is a non-stationary series and the variance of *Y*_*t*_ increases with time. If |*θ*| < 1, *Y*_*t*_ is a stationary series.

Thus, the hypothesis of stationarity can be evaluated by testing whether *θ* is strictly less than one, i.e., H_0_: *θ* = 1 (unit root in *θ* (*z*) = 0) ⇒*Y*_*t*_ ∼ *I* (1) *H*_1_: |*θ* | < 1 ⇒*Y*_*t*_ ∼ *I* (0).

The standard Dickey–Fuller test is conducted by estimating equation (8) after subtracting*Y*_*t*−1_ from both sides of the equation and obtaining the following equation:

(9) $$\begin{array}{c} \Delta Y_{t}=\alpha Y_{t-1}+\varepsilon_{t}, \end{array}$$

(10) $$\begin{array}{c} \varepsilon_{t}∼N\left[0,\sigma^{2}\right],\text{and }Cov\left[\varepsilon_{t},\varepsilon_{s}\right]=0\forall \;t\neq s, \end{array}$$

where*α*=*θ* − 1 andΔ*Y*_*t*_=*Y*_*t*_ − *Y*_*t*−1_ . The null and alternative hypotheses may be re-expressed as *H*_0_: *α* = 0 versus *H*_1_: *α* < 0 and evaluated using the conventional *t*-ratio:

(11) $$\begin{array}{c} t_{\alpha }=\frac{\hat{\alpha }}{s.e\left(\hat{\alpha }\right)}, \end{array}$$

where$\hat{\alpha }$ is the estimate of *α* and$s.e\left(\hat{\alpha }\right)$is the standard error of $\hat{\alpha }$. Equation (10) shows that, under the null hypothesis of a unit root, this statistic does not follow the conventional Student's t-distribution, and they derive asymptotic results and simulate critical values for various tests and sample sizes.

## 4. Results and Discussions

### 4.1. Unit Root Test Results

The results of the Augmented Dickey–Fuller and Phillips tests applied to the variables mentioned in the model of this study at level I (0) meaning testing the existence of unit root of the raw data as it is without differencing and first difference I (1) which means testing the existence of unit root of once differenced data are presented in Table 1.

The results reported in Table 1 indicate stationarity test of the variables at level form I (0). The null hypothesis of non-stationarity cannot be rejected even at ≤0.01 level for any of the variables because the critical values of Mackinnon test for ADF and PP are (−3.5) at ≤ 0.1; (−2.888) at ≤ 0.05; and (−2.578) at ≤ 0.01. To reject the null hypothesis, ADF and PP test statistics should be greater than the critical value, or in other words, the *P* value should be significant at specific level of confidence. Since the null hypothesis was not rejected for all the variables at any convenient significant level, all the variables had unit root at levels. Therefore, we can conclude that all the variables data are non-stationary at level.

From the results in Table 1, the Augmented Dickey–Fuller (ADF) and the Phillips–Perron (PP) test statistics for the first differences of all the series data were significant at 1% level of significance. This showed that the series data is stationary at first difference and hence the variables are considered as integrated of order one or I (1) process.

### 4.2. Ordinary Least Squares Analysis

In testing for the appropriate standard BLUE, ordinary least squares analysis was used. After testing the residual series for each specification for stationarity through the Augmented Dickey–Fuller and Phillis unit root tests, the outcomes of OLS results for pro-poor and pro-rich growth are stated in Tables 2 and 3.

Tables 2 and 3 are the results of the dynamic equation. The results from Table 2 show that all variables in the study were stationary at first difference. In the dynamic equation, the stationary levels of the variables are taken into consideration. Beginning with the poorest group in Table 2, the coefficients of the human capital, industrial, and services growth are negative. The coefficients of employment and agriculture growth are positive. As shown in Table 2, a total of 48.00% of variations in poorest volume are explained by the associations identified in this study. This statement is reflected by the R-squared value observed. In the richest income group, the coefficients of human capital, and industrial and service growths are positive. The coefficients of agricultural growth and employment creation are negative. About 56.00% of variations in richest volume are explained by the associations identified in this study.

From Tables 2 and 3, the variables in the study have positive and negative impacts on poorest and richest people. The results from Table 2 show that human capital, and industrial and services growth have negative impacts on poorest people, whereas employment and agriculture growth have positive impacts on poorest people. As shown in Table 3, in the richest income group, human capital, and industrial and service growths have positive impacts while agricultural growth and employment have negative impacts.

The data in the study is yearly data from 1990 to 2018; sample size is 28. To prove the results more, using more samples in detail is important. So, the quarterly data for the given yearly data was also analyzed and the descriptive result was stated as follows. The sources report the yearly data in the form of quarters. The quarterly data for 28 years was 112 quarters.

The empirical analysis included five series, namely, the human capital development (HC), growth in agriculture (AGR), total employment (EMP), industrial growth (INDG), and growth in services (GRS). Some descriptive statistics including the mean, the standard deviation, the coefficient of variation, and minimum and maximum values of the series under study are presented in Table 4. The results show that the values of summary statistics are more or less similar except standard deviation which indicates relatively high dispersion for growth in services.

### 4.3. Discussion of the Results

The study investigated the determinants of pro-poor growth and its impacts in Ethiopian economy between 1990 and 2018. Results presented in Tables 1–4 are interesting and met some degrees of expectation. My discussion will however concentrate on the dynamic results in Tables 2 and 3 because the analysis was based on stationary data.

The poor in every developing economy are found mainly in the agricultural sector. The 2008 Ethiopian agriculture survey showed that almost half of the farms in the country are family farms with annual income of less than the expected amount. Evidence shows that members of the family farm group own less than 2 ha of land and the poor control only 0.3% of the agricultural land area [1]. Table 2 shows that growth in agriculture has a positive effect on the income share of the bottom 20% group. The positive effect is in line with theoretical expectation that expansion in the sector where the poor carry out economic activity will bring an improvement in their income [15]. The finding falls in line with the work of [14, 21] which suggests that growth in agriculture has a positive effect on pro-poor growth. However, the growth in agriculture did not have a significant effect on the growth of the income of the poor in Brazil between 1990 and 2018.

Human capital development is important because it leads to skill development and helps the poor to acquire the necessary skill needed in the modern economy with better work condition. It is an expectation that improvement in the skill of the poor will give them opportunities to move to high paying jobs. In this study, human capital development led to a significant reduction in income share of the poor in Ethiopia between 1990 and 2018. This outcome is surprising but falls in line with the argument of the authors in [12] who maintained that the benefit of education spending in the developing countries always goes to the non-poor, and the authors in [16] pointed out that it is only the education spending at the basic level that is pro-poor.

Increases in employment opportunity expectedly had a positive and significant effect on pro-poor growth between 1990 and 2018. With a unit change in employment opportunity, that is, whenever employment increases by a unit, the income share of the poor from the economy's resources will increase by 0.09 units. It suggests that the more the jobs are created in Ethiopia, the more the income share of the poor in the country increases and the more the growth will be pro-poor. This suggests that increase in unemployment will have detrimental effect on pro-poor growth [28, 29]. As a consequence, the income shares of the poor fell and led to a reversal in pro-poor growth. Hence, for policy purpose, if government is interested in the reduction in poverty and inequality, government programmes must target creation of more jobs.

In real-life situations, the industrial sector is largely owned by the middle- and high-income groups. The implication is that the benefits of growth in the sector do not go direct to the poor but to the rich and the middle class who are the owners of the assets. However, if the link between agriculture and industrial sector is high, growth in the industrial sector can generate growth in the agriculture activities and the poor will benefit. The result of the dynamic equation in Table 2 shows that expansion in industrial activities has a negative but insignificant effect on the income share of the poor. Growth in the service sector has a negative and insignificant effect on the income share of the poor [30]. A unit increase in the service sector will reduce the income share of the poorest group.

Table 3 shows the dynamic results for the highest 20 percent income group. The richest group is included in the analysis only because we want to use it as a check on the result of the poor. In actual fact, the result turned out as expected. For instance, growth in the service sector had a significant effect on the increase in the income share of the highest 20 percent group. The factors which have positive effects on the income of the poor turned out to have negative effect on the income of the rich. The finding is very important for policy-making purpose. For example, in pursuance of social investment through tax policy, the best way to finance it so that it will not hurt the poor is to tax the service sector. It will lead to redistribution of resources from the rich to the poor.

## 5. Conclusions

The present study analyzed the determinants of pro-poor growth and its impacts on income share in Ethiopia using time series data from 1990 to 2018. In this study, the main determinants of pro-poor growth were used for analysis as the impacts of pro-poor growth. The results of the analysis showed that human capital, and industrial and services growth have negative impacts on the poorest people, whereas employment and agriculture growth have positive impacts on the poorest people. In the richest income group, human capital, and industrial and service growths have positive impacts while agricultural growth and employment have negative impacts.

The essence of going into the investigation on the determinants of pro-poor growth is because the world is interested in redistribution of benefits of growth in such a way that global poverty will fall. From the analyses with data from Ethiopia, employment was the significant factor that led to increase in the income share of the bottom 20% as well as the pro-poor growth in Ethiopia between 1990 and 2018. Government spending in education to build human capital significantly reduced the income share of the poor between 1990 and 2018. Moreover, the study reveals that increase in education spending and growth of the service sector had significant effect on the increase in the income share of the highest 20 group.

On the reverse of the pro-poor growth in Ethiopia in 2016, this is traced to increase in unemployment in the country in 2016. Therefore, to reverse back to pro-poor growth in Ethiopia, policy should target employment generation programmes. The policy direction of the result is that whenever government action leads to expansion in the service sector when every other thing remains the same, inequality will rise because the income share of the richest group will rise and the share of the poorest group will fall. Similarly, employment promotion programmes will narrow poverty and inequality in the country.

## Figures and Tables

**Table 1:** Results of ADF and PP unit root tests at level and at first difference.

Series	Level with intercept and trend				First difference with intercept and trend			
	Test statistic		Prob.^∗^		Test statistic		Prob.^∗^	
	ADF	PP	ADF	PP	ADF	PP	ADF	PP
LYS_H_	1.333	0.333	0.888	0.887	−3.833	−1.811	0.000^∗∗^	0.000^∗∗^
LHC	−3.131	−3.137	0.101	0.331	−1.303	−3.138	0.000^∗∗^	0.000^∗∗^
LIND	−3.183	−3.138	0.030	0.338	−3.833	−3.701	0.000^∗∗^	0.003^∗∗^
LAGR	−3.337	−3.073	0.303	0.130	−3.131	−3.133	0.000^∗∗^	0.001^∗∗^
LEMP	−3.138	−3.311	0.313	0.331	−3.783	−3.183	0.003^∗∗^	0.001^∗∗^
LSERV	−3.151	−3.331	0.033	0.313	−3.335	−3.531	0.000^∗∗^	0.001^∗∗^
YS_L_	−3.133	−3.181	0.133	0.133	−3.319	−3.331	0.001^∗∗^	0.003^∗∗^
Values of Mackinnon test for ADF and PP: 1% = −3.5								
3% = −3.888								
10% = −3.578								

**Table 2:** Dynamic equation (dependent variable = lowest 20% income group).

Variables	Coeff.	Std. error	t	Prob.
C	0.6854	0.0012	0.98	0.321
D (logHC)	−0.3297	0.1214	−2.15	0.023
D (IND)	−0.3612	0.3036	−0.18	0.781
D (AGR)	0.4125	0.5189	0.19	0.735
D (EMP)	0.09	0.0480	6.59	0.000
D (SERV)	−0.0034	0.0069	−1.45	1.971
*R* ^2^	0.48			
F-stat	12.56			
Prob	0.0000			

Source: analysis of Ethiopian data.

**Table 3:** Dynamic equation (dependent variable = highest 20% income group).

Variables	Coeff.	Std. error	t	Prob.
C	−0.0125	0.0965	−0.14	0.8954
D (logHC)	3.8941	1.5781	2.26	0.0213
IND	2.0198	0.3223	0.86	0.3698
AGR	−4.6218	0.2321	−1.27	0.2125
D (EMP)	−1.3950	0.1984	−7.99	0.0000
D (SERV)	0.2691	0.18912	2.59	0.0269
R2	0.56			
F-stat	17.38			
Prob	0.0000			

Source: analysis of Ethiopian data.

**Table 4:** Descriptive statistics for quarterly data.

Series	Obs.	Mean	Std. dev.	Min	Max	CV
HC	112	253.251	68.235	12.130	303.260	0.50168
IND	112	265.023	58.261	19.212	512.0362	0.362
AGR	112	269.241	74.231	24.102	263.235	0.962
EMP	112	256.120	59.241	10.321	246.210	0.425
SERV	112	213.256	51.321	12.320	412.096	0.546

## Data Availability

The data used to support the study are available from the author upon request.
